# Defining an olfactory receptor function in airway smooth muscle cells

**DOI:** 10.1038/srep38231

**Published:** 2016-12-01

**Authors:** William H. Aisenberg, Jessie Huang, Wanqu Zhu, Premraj Rajkumar, Randy Cruz, Lakshmi Santhanam, Niranjana Natarajan, Hwan Mee Yong, Breann De Santiago, Jung Jin Oh, A-Rum Yoon, Reynold A. Panettieri, Oliver Homann, John K. Sullivan, Stephen B. Liggett, Jennifer L. Pluznick, Steven S. An

**Affiliations:** 1 Department of Physiology, Johns Hopkins School of Medicine, Baltimore, MD 21205, USA; 2 Department of Environmental Health Sciences, Johns Hopkins Bloomberg School of Public Health, Baltimore, MD 21205, USA; 3 Department of Anesthesiology and Critical Care Medicine, Johns Hopkins School of Medicine, Baltimore, MD 21205, USA; 4 Institute for Translational Medicine and Science, Rutgers University, New Brunswick, NJ 08901, USA; 5 Genome Analysis Unit, Amgen Inc., South San Francisco, CA 94080, USA; 6 Department of Inflammation, Amgen Inc., Thousand Oaks, CA 91320, USA; 7 Department of Internal Medicine and Molecular Pharmacology and Physiology, and the Center for Personalized Medicine and Genomics, University of South Florida Morsani College of Medicine, Tampa, FL 33612, USA; 8 Department of Chemical and Biomolecular Engineering, Johns Hopkins University, Baltimore, MD 21205, USA; 9 Department of Biomedical Engineering, Ulsan National Institute of Science and Technology, Ulsan 689-798, Republic of Korea

## Abstract

Pathways that control, or can be exploited to alter, the increase in airway smooth muscle (ASM) mass and cellular remodeling that occur in asthma are not well defined. Here we report the expression of odorant receptors (ORs) belonging to the superfamily of G-protein coupled receptors (GPCRs), as well as the canonical olfaction machinery (G_olf_ and AC3) in the smooth muscle of human bronchi. In primary cultures of isolated human ASM, we identified mRNA expression for multiple ORs. Strikingly, *OR51E2* was the most highly enriched OR transcript mapped to the human olfactome in lung-resident cells. In a heterologous expression system, OR51E2 trafficked readily to the cell surface and showed ligand selectivity and sensitivity to the short chain fatty acids (SCFAs) acetate and propionate. These endogenous metabolic byproducts of the gut microbiota slowed the rate of cytoskeletal remodeling, as well as the proliferation of human ASM cells. These cellular responses *in vitro* were found in ASM from non-asthmatics and asthmatics, and were absent in *OR51E2*-deleted primary human ASM. These results demonstrate a novel chemo-mechanical signaling network in the ASM and serve as a proof-of-concept that a specific receptor of the gut-lung axis can be targeted to treat airflow obstruction in asthma.

Asthma is a chronic airway inflammatory disease that afflicts over 300 million individuals worldwide^1^. The major cause of morbidity and mortality of the disease is airflow obstruction, which is in part due to actively constricted smooth muscle of human bronchi, as well as increased smooth muscle mass^2^,^3^,^4^. Therapies for airflow obstruction from airway smooth muscle (ASM) contraction include the use of antagonists to receptors for specific spasmogens that are released locally due to inflammation or other triggers, or β-agonists, which are direct bronchodilators acting on ASM β_2_-adrenergic receptors (β_2_ARs)^5^,^6^. Both approaches utilize G-protein coupled receptors (GPCRs) as targets, and indeed GPCRs are extensively expressed on human ASM^7^.

We have undertaken studies of this superfamily of receptors^8^ to identify new targets for treating the contraction and proliferative phenotypes of ASM in asthma. Unexpectedly, we^9^ and others^10^ have found expression of multiple bitter taste ‘sensory’ GPCRs (TAS2Rs) in the lung, in particular motile cilia of airway epithelium and ASM of human bronchi. In these lung-resident cells, similar to type II taste receptor cells on the tongue^11^, activation of TAS2Rs mediates the coupling of the gustducin G protein (G_gust_) to phospholipase Cβ (PLCβ) and evokes an increase in intracellular calcium ([Ca^2+^]_i_). In motile cilia, this rise in [Ca^2+^]_i_ enhances ciliary beat frequency and, hence, is posited to play a role in mucus clearance^10^. In ASM, TAS2R-mediated increases in [Ca^2+^]_i_, and contrary to the classical excitation-contraction mechanisms^12^, cause membrane hyperpolarization, ASM relaxation, and bronchodilation^9^. TAS2Rs are broadly tuned, and bitter compounds of diverse structures are effective in interdicting the muscle’s mechanical force on the airway via an entirely different cascade of signals compared to conventional β-agonists^13^,^14^,^15^. Certain bacteria are known to secrete substances that act as TAS2R agonists, which may be the evolutionary basis for expression of these receptors on cilia and ASM, acting to increase mucous clearance and airway opening, respectively.

In the last decade, a number of the other sensory GPCRs (i.e. visual, taste and odorant receptors) that were long thought to be specific to classic sensory physiology have been found on other tissues, where they serve as selective and sensitive photo/chemoreceptors^16^,^17^,^18^,^19^,^20^. For example, a non-image-forming opsin (Opn4) is found in the smooth muscle lining blood vessels, where it promotes light-dependent vasorelaxation^20^. Olfactory-like chemosensory signaling is found in a variety of tissues outside of the main olfactory epithelium^19^,^21^,^22^ and is reported to play diverse homeostatic roles. These include sperm chemotaxis^23^, muscle cell migration^24^, renin secretion and blood pressure control^25^,^26^, and ventilation in response to hypoxia^27^. Although the selective pressures or evolutionary context that have led to these receptors being expressed in non-sensory tissues is not entirely clear, these findings have now given rise to the notion that agents acting upon these receptors may represent a previously unrecognized sensory system that may provide for novel therapeutic targets. Given that olfactory receptors (ORs) are the largest family of GPCRs in the superfamily^28^, and can detect and discriminate volatile environmental chemicals over a wide odorant space^29^, here we applied molecular-screening, whole-transcriptome and single-cell analyses to interrogate the expression landscape of ORs in human ASM cells with the goal of identifying receptors that might promote or modify the asthmatic phenotype. The results of these studies identified a physiologic function of OR51E2 that could be exploited further for treatment of obstructive lung disease by decreasing ASM remodeling and muscle mass.

## Results

### Novel olfactory-like chemosensory network in human ASM cells

**Figure 1:**
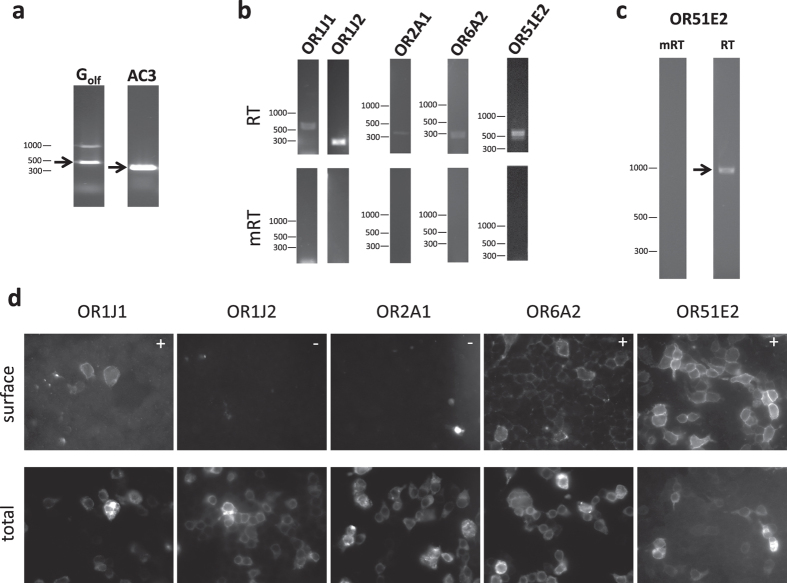
Expression of olfactory receptor mRNA transcripts in human ASM and transfected HEK-293T cells. (**a**) G_olf_ and AC3 are both expressed in human ASM cells (sequenced to confirm). (**b**) The five ORs transcripts identified by degenerate PCR were also confirmed in human ASM, using gene-specific primers to each OR. Mock RT samples (below) serve as negative controls. (**c**) Confirmed expression of the full-length transcript of OR51E2 in RT, but not in mRT. (**d**) Three of the five ASM ORs are able to traffic to the surface of HEK-293T cells. The top panel shows cell surface OR population as detected by immunostaining against an N-terminal extracellular FLAG epitope tag with a polyclonal antibody. For detecting the internal OR population, cells were subsequently fixed, permeabilized, and stained again for Flag using a monoclonal antibody (bottom panel). All constructs with the exception of OR51E2 contain a leucine rich N-terminal signal peptide and are co-transfected with chaperon proteins as previously described^26^,^34^; “+” and “−” indicate cell surface localization of ORs.

Olfactory sensory neurons (OSNs) in the main olfactory epithelium can detect and discriminate volatile chemicals of a wide odorant space^29^. Each of the hundreds of mammalian ORs expressed in individual OSNs signals through a single common pathway: the olfactory G protein (G_olf_) and the olfactory form of adenylyl cyclase III (AC3)^30^,^31^. Hence, we initially probed for transcript levels of these obligate constituents of the olfactory machinery in isolated human ASM cells by RT-PCR. As shown in Fig. 1a, primary human ASM cells in culture expressed both G_olf_ and AC3. These RT-PCR products were confirmed by sequencing and found to be indistinguishable from previously published sequences from the olfactory epithelium.

In order to detect potential ORs expressed in ASM, we then utilized a PCR approach employing degenerate primers capable of amplifying the entire repertoire of ORs, as we and others have documented previously^19^,^32^. Using this low stringent strategy, followed by sequencing of the PCR products, we identified 4 different ORs (OR1J1, OR2A1, OR6A2, and OR51E2) and one OR pseudogene OR2A20P. We confirmed the presence of each candidate ASM OR using RT-PCR with gene-specific primers (Fig. 1b and Supplementary Fig. 1). As shown in Fig. 1b, mRNA encoding OR1J2 (highly similar to OR1J1) was also found to be present in human ASM. These results demonstrate that human ASM cells express multiple ORs and their recognized downstream signaling components.

### Heterologous expression of human ASM ORs

We next cloned and sequenced all identified ASM ORs, and ectopically expressed each of the full-length OR mRNAs in HEK-293T cells (Fig. 1c,d). We conducted these cell surface trafficking studies because most ORs are found to be trapped in the endoplasmic reticulum when transfected into model cells^33^, and thus the majority of ORs remains orphan receptors. As shown in Fig. 1d, Flag-tagged full-length constructs encoding OR1J1, OR6A2 and OR51E2 were able to traffic to the cell surface under certain conditions; surface expression of OR51E2 was the most pronounced among the three ORs. In fact, OR51E2 incorporated into the plasma membrane without the aid of an N-terminal leucine-rich cleavable signal peptide (Lucy tag, as previously described^34^), or co-expression of RTP1S and Ric8b chaperones (Fig. 1d). In contrast, the surface expression of OR1J1 and OR6A2 required the Lucy tag and co-expression of the chaperone proteins (Fig. 1d). However, even with the Lucy tag and the chaperones, OR1J2 and OR2A1 failed to reach the plasma membrane (Fig. 1d).

### Human body atlas of ASM ORs

**Figure 2:**
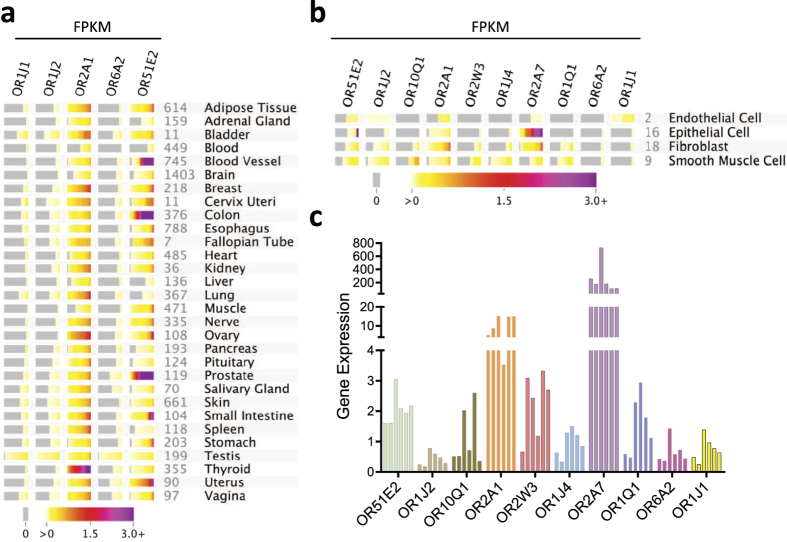
Human body atlas of ASM OR. (**a**) Expression of the five *de novo* identified ASM ORs across 30 human tissue types (GTEx RNA-Seq tissues). Each colored block provides a quantile spread representing expression levels expressed as fragments per kilobase of transcript per million mapped reads (FPKM) across all samples of that tissue type. The color at the middle of the strip represents the median expression, and the color at the end of the strip represents the maximum expression. A complete absence of reads over an OR is represented as grey. The number of human samples in the collection is displayed next to the tissue name. (**b**) Cultured lung cell expression of the indicated ORs (also see Supplementary Table 1). (**c**) RT-PCR cross-validation of the most abundant lung-resident OR genes (shown in panel b) in human ASM cells isolated from 6 non-asthmatic lung donors. Results are shown as 2^−ΔCt^ X 10000, where ΔCt = [Ct(target gene) − Ct(gapdh)].

In order to validate the expression results, we ascertained the expression of these *de novo* identified ASM ORs (OR1J1, OR1J2, OR2A1, OR6A2, and OR51E2) in the lung and other human organs. First, we surveyed RNA-Seq data from the Genotype-Tissue Expression Project (GTEx) which profiled 30 different tissue types^35^. In this dataset, transcripts for the ASM ORs (OR1J1 and OR1J2 encoded on chromosome 9; OR2A1 encoded on chromosome 7; and OR6A2 and OR51E2 encoded on chromosome 11) were noted to be expressed in multiple tissues, albeit at low levels (Fig. 2a), and were also found in a subset of immune cells (Supplementary Fig. 2; RNA-Seq immune cell dataset from the BLUEPRINT project). As expected, ASM ORs were also reported in whole lung tissue (Fig. 2a). In order to further map these sequence data to other structural cell-types of the lung, we then utilized an RNA-Seq lung cell dataset comprising cultured epithelial, endothelial, fibroblast and smooth muscle cells (Amgen, Inc., Thousand Oaks, CA). Three of the ASM *de novo* identified ORs (OR51E2 > OR1J2 > OR2A1) were among the most highly enriched OR transcripts in these cell types (Supplementary Table 1). The other two ORs (OR6A2 and OR1J1) were detected, but at very low abundance (Supplementary Table 1 and Fig. 2b). Of note, while multiple OR genes/pseudogenes are encoded and clustered in close proximity of OR51E2 on chromosome 11, robust expression of only OR51E2 was detected across lung-resident cells (Supplementary Fig. 3). Using RT-PCR, we confirmed the expression profile of the most abundant lung-resident ORs (Fig. 2b) in human ASM cells isolated from multiple lung donors (Fig. 2c).

### OR51E2 activation modulates cytoskeletal remodeling in ASM

**Figure 3:**
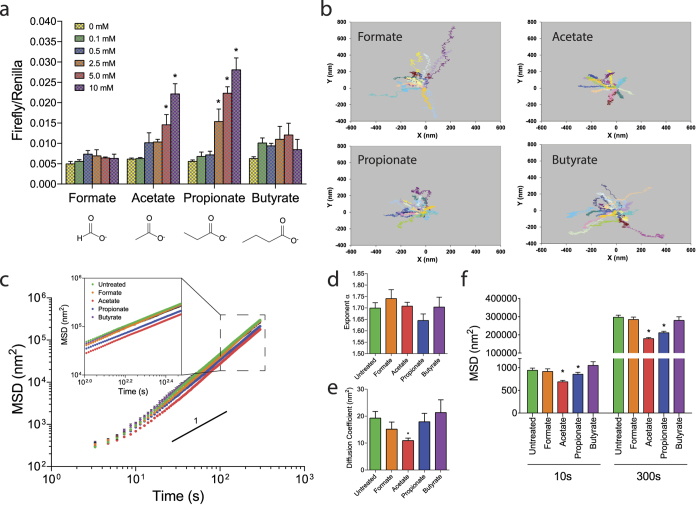
De-orphanization of OR51E2 signaling and function in human ASM. (**a**) Agonist profiles of OR51E2 as detected by a luciferase-based reporter assay in transfected HEK-293T cells. For each ligand dosage/condition (n = 3), we used ANOVA followed by the Student-Newman-Keuls method to determine significance. Data are presented as mean ± SE (*P < 0.05 vs. 0 mM dose considered significant). (**b**) Trajectories of unforced motions of RGD-coated beads functionalized on the living human ASM. Spontaneous bead motions were recorded over the course of 5 min (10X Objective). Cells were treated for 24 h with or without 10 mM of the respective SCFAs (formate, acetate, propionate, and butyrate). For clarity, only a few representative tracings (n = 20) are depicted for each experimental conditions. (**c**) The trajectories of bead motions in two dimensions were characterized by computed mean square displacement (MSD) of all beads as function of time *t*. Data are presented as mean ± SE (untreated, n = 15; formate, n = 9; acetate, n = 15; propionate, n = 17; butyrate, n = 5 individual cell-wells, comprising ~442-1874 individual beads measurements). (**d,e**) For each individual cell-well, the exponent α and the diffusion coefficient (D*) were estimated from a least square fit of a power law. (**f**) MSD measured at 10 s and 300 s, respectively. *P < 0.05 compared with untreated cells by ANOVA and post hoc t-tests.

Ligands for sensory receptors are often generated by essential physiological processes, such as fermentation of non-digestible polysaccharides by the gut microbiota^26^,^36^,^37^,^38^,^39^. Toward this end, OR51E2 and its murine ortholog (Olfr78) have been reported to respond to metabolic byproducts of anaerobic bacterial fermentation, including short chain fatty acids (SCFAs) and lactate^26^,^27^. First we ascertained the agonist activation profile of OR51E2 expressed in HEK-293T cells to define agonists for use in ASM cells. We used a luciferase-based reporter assay in which OR-ligand binding evokes increased intracellular cyclic-AMP (cAMP) that in turn drives a cAMP response element-dependent expression of luciferase. As shown in Fig. 3a, while formate and butyrate had no effect, acetate and propionate increased luciferase expression in a concentration-dependent manner, consistent with the reported receptor-ligand pairing of OR51E2/Olfr78 in the kidney^26^. Recently, Chang and colleagues^27^ reported lactate as a ligand for the murine ortholog of OR51E2 (Olfr78). Indeed, we found that Olfr78 responded to lactate–albeit with a higher EC_50_ than previously reported (Supplementary Fig. 4a). Lactate failed to activate OR51E2 within the physiological range of lactate concentrations, however (Supplementary Fig. 4b). Thus, we undertook mechanistic studies to ascertain the cellular function of OR51E2 in isolated ASM cells using the metabolic byproducts of anaerobic bacterial fermentation, acetate and propionate, that evoked the aforementioned second messenger response.

Because OR51E2 signals through G_olf_ and AC3, and because β_2_AR relax ASM by generating cAMP^15^, we first probed dynamic changes in ASM stiffness in response to a panel of SCFAs using magnetic twisting cytometry (MTC). In this method, we applied forced motions of a functionalized bead tethered to the underlying cytoskeleton through the cell surface integrin receptors. Dynamic changes in the stiffness measured with this single-cell technology are robust indices for contraction and relaxation of isolated ASM cells^4^,^9^. Unlike bitter tastants of diverse structures that caused rapid and substantial decreases in the stiffness of isolated human ASM cells^9^, none of the SCFAs in the range typically detected in the digestive tract, serum and/or the lung (0.1–10 mM^38^,^40^,^41^,^42^) or in the range of detection of cAMP signaling of OR51E2 (EC_50_ = ~2 mM), caused acute changes in the cell stiffness (data not shown), nor did they alter histamine-induced single-cell contraction (Supplementary Fig. 5a). Consistent with the absence of a rapid relaxation effect, we were unable to detect an increase in intracellular cAMP in ASM cells from acetate or propionate exposures of 30 min and 4 h (data not shown) using a sensitive immunofluorescence assay which we have previously utilized for isolated ASM^43^. This was true even when phosphodiesterase inhibitors were added to the media (data not shown). We noted, however, appreciable decreases in baseline cell stiffness with prolonged (24 h) exposures to acetate and propionate (Supplementary Fig. 5b). This delayed response is not typical of a cAMP promoted, PKA dependent, relaxation as is found with β_2_AR agonists, suggesting another mechanism of action. These results prompted us to explore, in time and space, other mechanical/physiological correlates that might be affected by SCFAs, including the rate of cytoskeletal remodeling and cellular proliferation.

As shown in Fig. 3b, 24 h exposures to acetate and propionate, but not formate and butyrate, decreased spontaneous nanoscale motions of individual functionalized beads on human ASM cells. We characterized these unforced bead motions by computing the mean square displacements (MSDs) of all beads as a function of time *t*. For all conditions, computed MSDs of unforced beads increased with *t* as a power law with an exponent α greater than unity (Fig. 3c,d). As such, these anomalous motions are non-thermal in nature^44^, and represent a discrete molecular-level rearrangement of the underlying cytoskeletal network that is driven by an additional source of energy in the living cell^45^,^46^, a phenomenon termed cytoskeletal remodeling. Compared with untreated human ASM cells, those treated with acetate and propionate, but not formate or butyrate exhibited decreases in the computed MSDs, which are apparent at times >10 s and up to 300 s (Fig. 3c–f). These decreases in MSDs in response to acetate and propionate were dose dependent (Supplementary Fig. 5c,d). There were no statistical differences in the super-diffusive exponent α across the groups (Fig. 3d). Interestingly, cells treated with acetate showed an appreciable decrease in the diffusion coefficient (D*), which is a measure of the incremental step of spontaneous bead motion (Fig. 3e). None of the other SCFAs, including propionate, significantly altered D* as compared to that of untreated cells.

### OR51E2/Olfr78 is the chemo-mechanical receptor for the SCFAs acetate and propionate in ASM

To ascertain if the discrete cytoskeletal remodeling events induced by acetate and propionate are mediated via OR51E2 in human ASM cells, we next applied the clustered regularly interspaced short palindromic repeats (CRISPR) and CRISPR-associated protein 9 (Cas9) to perform loss-of-function studies. Consistent with results shown above (Fig. 3), in mock transfected primary human ASM cells, 24 h exposures to acetate and propionate decreased cytoskeletal remodeling dynamics (Supplementary Fig. 6). In *OR51E2*-edited human ASM cells, propionate decreased the rate of cytoskeletal remodeling while there was no effect from acetate (Supplementary Fig. 6). We were not able to definitively validate the efficiency of RNA-guided endonucleases in deleting OR51E2 protein in the primary human ASM, because of the poor sensitivity of the commercially available antibodies for OR51E2. This difficulty and the lack of a loss of responsiveness to acetate in OR51E2-edited human ASM cells prompted us to study the OR51E2 murine ortholog *Olfr78.* This approach provided for use of a highly sensitive and validated *Olfr78* antibody and to examine several inbred mouse strains.

**Figure 4:**
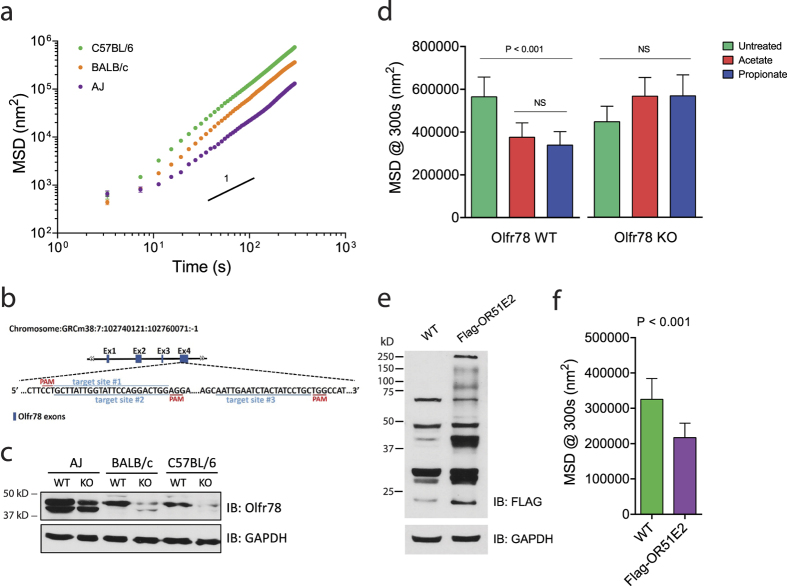
Characteristics of the OR51E2 homologue Olfr78. (**a**) Cytoskeletal dynamics of ASM cells derived from several inbred mouse strains as measured by SNTM. Data are presented as mean ± SE (AJ, n = 825; BALB/c, n = 599; C57BL/6, n = 805 individual beads measurements). (**b**) A schematic diagram of murine *Olfr78* locus and its exons; three target sites in exon 4 are shown below, indicating the CRISPR N20NGG target sites. (**c**) Genome editing efficiency of CRISPR-Cas9 in the primary mouse ASM cells as determined by western blot. Full-length gels/blots are presented in Supplementary Figure 7a. (**d**) Computed MSD at 300 s for Olfr78 WT and Olfr78 KO cells (C57BL/6) in response to 24 h exposures to acetate and propionate (10 mM). Data are presented as geometric mean ± 95% CI (n = 307–379 individual beads measurements for each group). (**e**) Isolated mouse ASM cells (C57BL/6) were transfected with or without FLAG-tagged full-length construct encoding human OR51E2. OR51E2 protein expression was detected by anti-Flag antibody. Full-length gels/blots are presented in Supplementary Figure 7b. Although the expected band is ~36 kDa, ORs typically also appear as multiple higher molecular weight bands^34^. (**f**) Computed MSD at 300 s for wild-type (WT) and OR51E2-overexpressing (Flag-OR51E2) mouse ASM cells. Data are presented as geometric mean ± 95% confidence interval (n = 354–364 individual beads measurements for each group).

As shown in Fig. 4a–c, we designed guide RNAs to target Cas9 to the exon 4 of *Olfr78* gene in primary ASM cells isolated from three different mouse strains (AJ, BALB/c, and C57BL/6). Under basal conditions, we noted differential protein expression levels of Olfr78 in isolated mouse ASM cells: AJ cells exhibited the highest Olfr78 expression, followed by BALB/c and C57BL/6 (Fig. 4c and Supplementary Fig. 7a). Across the three primary mouse ASM cells, however, CRISPR-Cas9 successfully reduced Olfr78 protein expression with robust decreases particularly noted in BALB/c and C57BL/b ASM cells. In WT C57BL/6 cells, 24 h exposures to acetate and propionate decreased the dynamics of cytoskeletal remodeling (Fig. 4d). Compared with untreated cells, the rate of cytoskeletal remodeling was significantly reduced in cells treated with acetate and propionate. In *Olfr78*-edited cells, however, the dynamics of cytoskeletal remodeling were unaffected by both acetate and propionate. Taken together, these results in isolated human and mouse ASM establish that acetate and propionate are agonists for OR51E2/Olfr78 and that such olfactory-like chemosensory signal transduction affects the dynamics of the underlying cytoskeletal network in ASM.

It is interesting to note that AJ ASM cells, which intrinsically expressed the highest Olfr78 protein, exhibited the least degree of cytoskeletal remodeling compared to the other strains (Fig. 4a) and showed no further decreases in the remodeling in response to acetate and propionate (data not shown). We speculate this may be due to spontaneous activation of the unoccupied receptors and, hence, the saturation of signal transduction^47^,^48^. In support of this notion, overexpression of human OR51E2 in isolated C57BL/6 mouse ASM cells caused appreciable slowing of the cytoskeletal remodeling in the absence of the agonists (Fig. 4e,f and Supplementary Fig. 7b).

### Activation of OR51E2 inhibits human ASM proliferation

**Figure 5:**
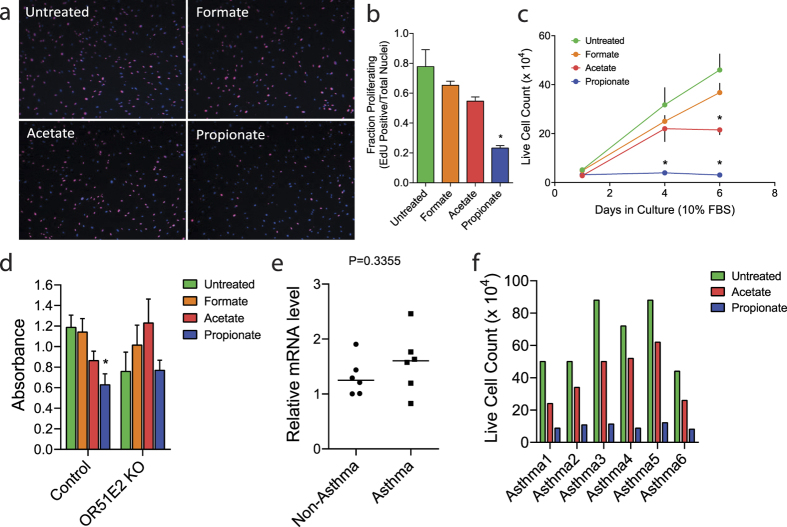
Activation of OR51E2 inhibits ASM proliferation. (**a**) Representative photomicrographs of cells treated for 24 h with or without formate, acetate, and propionate (10 mM) as measured by EdU cell proliferation assay. EdU positive nuclei are labeled with Alexa Fluor 647 (purple), and all nuclei are labeled with DAPI (blue). (**b**) The ratio of Alexa Fluor 647 positive and DAPI positive nuclei were counted in four non-overlapping fields per sample. Data are presented as mean ± SE (n = 3 independent samples). (**c**) ASM proliferation was measured by live cell counting over 6 days in culture with SCFAs. Data are presented as mean ± SE (n = 4 independent measurements). (**d**) The effects of SCFAs on cellular proliferation of WT and OR51E2 KO cells as quantified by Cell Counting Kit-8 (CCK-8, Sigma). For these studies, cells were treated for 24 h with 10 mM SCFAs and then 1 mM SCFAs for the next 5 days in culture. CCK-8 assay was performed on day 6. Data are presented as mean ± SE (n = 3 independent measurements). (**e**) The relative transcript levels of OR51E2 in isolated human ASM (non-asthmatics, n = 6 lung donors; asthmatics, n = 6 lung donors). (**f**) The effects of SCFAs, acetate and propionate, on cellular proliferation of asthmatic ASM derived from 6 independent lung donors as measured by live cell counting over 6 days in culture.

In addition to the cytoskeletal effects, we found that acetate and propionate inhibited cellular proliferation of isolated human ASM cells in culture (Fig. 5). Here, propionate appeared to be more effective than acetate, and indeed a 24 h exposure to propionate was sufficient to inhibit DNA synthesis and substantially depress cellular proliferation (Fig. 5a,b). By 6 days in culture, both acetate and propionate significantly attenuated ASM proliferation (Fig. 5c). These inhibitory effects of acetate and propionate were absent in *OR51E2*-edited primary human ASM cells (Fig. 5d). We also noted that human ASM expressed GPCRs of the GPR family (GPR41 and GPR43), albeit at substantially lower expression levels than OR51E2 (Supplementary Table 1 and Supplementary Fig. 8a). GPR41 and GPR43 exhibit ligand profiles similar to that of OR51E2^26^,^36^,^37^,^38^,^39^. As shown in Supplementary Figure 8b–e, however, shRNA-mediated knockdown of GPR41 or GPR43 did not attenuate either acetate- or propionate-induced inhibition of ASM proliferation in culture. Collectively, these results demonstrate that an additional chemoreceptor effect of OR51E2 expressed in ASM for the agonists acetate and propionate is a prolonged inhibition of cellular proliferation.

### OR51E2 mitigates increased ASM mass in asthmatic ASM cells

A cardinal feature of airway remodeling in asthma is increased ASM mass^2^, which is a combination of hypertrophy and hyperplasia. And indeed, cultured human ASM cells derived from asthmatics have an increased proliferation rate compared to non-asthmatic cells^49^. Hence, we assessed the effects of acetate and propionate to inhibit the proliferation of ASM cells derived from asthmatics in culture. For these studies, we used human ASM cells prepared from six independent asthmatic lung donors. As shown in Fig. 5e, we detected a variable level of OR51E2 mRNA between ASM lines derived from different individuals. There was no statistical difference in OR51E2 mRNA between asthmatic and non-asthmatic cells. Importantly, similar to non-asthmatic cells, both acetate and propionate effectively inhibited cellular proliferation of asthmatic ASM in culture (Fig. 5f). These findings suggest that OR51E2 is intact and operational in ASM from asthmatic lungs, and that its ligands, acetate and propionate, could potentially mitigate increased ASM mass associated with asthma pathology.

## Discussion

Here we demonstrate expression of odorant receptors in primary human ASM cells. ORs that are expressed on human ASM were also found in a variety of human tissues, including other structural cells of the lung. OR51E2 was expressed in multiple lung-resident cells, including the smooth muscle of human bronchi. Transfected OR51E2 trafficked readily to the cell surface of HEK-293T cells and showed dose-dependent responsiveness to the SCFAs acetate and propionate, but not butyrate or formate. In ASM, acetate and propionate caused reductions in the rate of cytoskeletal remodeling as well as cellular proliferation. These physiologic effects were mediated via OR51E2 as indicated by loss of function in gene-edited cells. OR51E2 expression was not altered in ASM cells derived from asthmatic subjects compared to non-asthmatic, and activation depressed cellular proliferation of the asthmatic ASM cells.

Interestingly, OR51E2 activation did not result in acute ASM relaxation, which might be expected since in olfactory epithelium the signal transduction of OR51E2 and other olfactory receptors is linked to cAMP production. And indeed, we observed dose dependent increases in cAMP (as assayed by a luciferase reporter) in OR51E2 transfected HEK-293T cells. However, consistent with the lack of acute relaxation, we were unable to measure an increase in whole-cell cAMP in human ASM. This may be due to compartmentalization of cAMP signaling by OR51E2 and thus a negligible increase in total intracellular cAMP. Alternatively, OR51E2 could couple to other G-proteins or effectors in ASM leading to the observed physiological effects. Indeed, many GPCRs that were at one time thought to couple to one G-protein/pathway have now been shown to couple to other G-proteins, or even to non G-protein pathways^48^. This “multidimensional” signaling can be highly cell-type dependent^48^, which often calls for studying a given receptor in the cell-type of interest, as we have carried out in the current study. Indeed, our studies with TAS2Rs in ASM also show a divergence from the classical signaling pathway described for this receptor in sensing bitter tastants in taste buds^9^. In taste cells, TAS2R activation by agonists causes cell membrane depolarization due to coupling to a transient receptor potential channel. In contrast, activation of ASM TAS2Rs by the same agonists causes membrane hyperpolarization, highlighting the cell-type specific nature of GPCR signaling events. In the current study, we are confident that the responses we have observed to known OR51E2 agonists in human ASM are in fact due to this receptor being activated because of the loss of physiologic function when the OR51E2 gene was deleted. The two functions that we have identified in ASM that result from OR51E2 activation are consistent with structural and physiological events that would become most apparent with chronic activation of the receptor. Both a decrease in molecular-level cytoskeletal rearrangement activity (remodeling), and cell proliferation, would act to suppress the increased ASM mass observed in asthma and thus mitigate airway obstruction in the disease.

In primary human ASM cells in culture, acetate and propionate effectively interdicted cellular proliferation. Previously, Fu and colleagues^50^ have reported that SCFAs inhibit the proliferation and migration of a human colonic adenocarcinoma cell line. In addition, SCFAs are known to inhibit T lymphocyte proliferation^51^ and modulate immune inflammatory responses^38^. In the gut mucosa, these suppressive effects of SCFAs on cellular proliferation and inflammation are thought to act through GPR41 and/or GPR43. In ASM, however, we found low transcript abundance of GPR41/43 and, moreover, shRNA-mediated knockdown of both GPR41 and GPR43 did not attenuate either acetate- or propionate-induced inhibition of ASM proliferation in culture.

SCFAs are endogenously generated by fermentation of undigested dietary fibers by the resident anaerobic bacteria in the gut^41^. They are essential energy sources for the epithelial lining of the gastrointestinal tract and are potent growth stimulators for the gut microbiota, including *Bifidobacterium*^52^. Trompette and colleagues^53^ have recently reported that dietary fermentable fiber content influence not only the richness and diversity in the microbiota of the gut, but also shape the lung microbiota. In mice fed a high-fiber diet, the authors also found increased circulating levels of SCFAs and demonstrated, in turn, reduced allergic immune inflammatory responses in the lung in animals treated with acetate or propionate. The authors posited, therefore, that these metabolic byproducts of gut (and possibly lung microbiota) might influence the observed reduction in allergic airway inflammation and airway hyper-reactivity^53^. Notably, SCFAs produced by the gut microbiota are absorbed into the bloodstream of the host and, thereby, they would be readily able to reach smooth muscle lining the vasculature, as well as the airways. Here we provide a new, non-immune mechanism in the gut-lung axis, where SCFAs directly activate olfactory receptors expressed on ASM to reduce ASM mass. It is also possible that resident lung microbiota may directly produce SCFAs in disease and/or health, although to our knowledge this has not yet been directly demonstrated. Taken together, these studies support the long-held view of the beneficial effects of dietary fibers and their fermentable SCFAs in various metabolic syndromes, including obesity which is a risk factor for asthma^54^.

Our data identifying an olfactory receptor-based signaling network on ASM has implications for elucidating the role of SCFAs, and gut or lung microbiota, in modulating the asthma diathesis. In addition, these data point to a new receptor target for potential therapeutics for asthma. Based on our current data, such agents would be agonists for OR51E2, which could act to inhibit or reverse the increased ASM mass and its effects on airflow obstruction observed in the disease.

## Methods

### Materials

Unless otherwise noted, reagents were obtained from Sigma-Aldrich with the exception of DMEM-Ham’s F-12 (1:1) which was purchased from GIBCO (Grand Island, NY). The synthetic arginine-glycine-aspartic acid (RGD) containing peptide was purchased from American Peptide Company (Sunnyvale, CA).

### Cell culture

As described elsewhere^9^, primary human ASM cultures were established from the proximal airways (first through third order bronchi) of deceased donor lungs unsuitable for transplantation in accordance with of the Institutional Review Boards at the University of Pennsylvania. Mouse ASM cells were prepared from the isolated trachealis of AJ, BALB/c and C57BL/6 mice as described^55^. All mice (male, 8-wk old) were obtained from a commercial supplier (Jackson Laboratory, Bar Harbor, ME) and housed in a conventional animal care facility at the Johns Hopkins Bloomberg School of Public Health (Baltimore, MD). The animal protocols were approved by the Institutional Animal Care and Use Committee for the Johns Hopkins University, and complied with Federal and State regulations governing the humane care and use of laboratory animals. Both mouse and human ASM cells were maintained in serum-free media for 24 h at 37 °C in humidified air containing 5% CO_2_ prior to study. These conditions have been optimized for seeding cultured cells on collagen matrix and for assessing their mechanical properties^4^,^9^,^55^.

### Live cell micromechanical methods

Using spontaneous and forced motions of RGD-coated ferrimagnetic microbeads (~4.5 μm in diameter) anchored to the cytoskeleton through cell surface integrin receptors of the adherent living cell, we detected cytoskeletal remodeling dynamics and the cell stiffness in response to a panel of SCFAs, respectively. These methods, spontaneous nanoscale tracer motions (SNTM) and magnetic twisting cytometry (MTC) are described in detail elsewhere^45^,^46^,^55^,^56^. For these studies, ASM cells were plated at 30,000 cells/cm^2^ on collagen-coated plastic wells (96-well Removawell, Immulon II, Dynetech). First, using SNTM, we visualized spontaneous nanoscale displacements of an individual functionalized bead (~50–100 beads per field-of-view) and recorded its positions at a frequency of 12 frames/s for *t*_max_ ~300 s via a CCD camera (Orca II-ER, Hamamatsu, Japan) attached to an inverted optical microscope (Leica Microsystems, Bannockburn, IL). The trajectories of bead motions in two dimensions were then characterized by computing the mean square displacement of all beads as function of time [MSD(t)] (nm^2^), as previously described^45^. Herein, we analyzed MSD data for times greater than 10 s and up to 300 s. For individual cells and experimental conditions, diffusion coefficient D* and the exponent α of the bead motion were also estimated from a least-square fit of a power-law to the average of MSD data versus time.

We then applied forced motions of the functionalized beads using MTC^4^,^9^,^55^,^56^ and measured the stiffness of individual ASM cells. In brief, RGD-coated ferrimagnetic beads bound on the surface of the cell were magnetized horizontally with a brief 1,000-Gauss pulse and twisted in a vertically aligned homogeneous magnetic field (20 Gauss) that was varying sinusoidally in time. This sinusoidal twisting magnetic field caused both a rotation and a pivoting displacement of the bead: as the bead moves, the cell develops internal stresses which in turn resist bead motions^56^. Lateral bead displacements in response to the resulting oscillatory torque were detected with an accuracy of 5 nm using an intensity-weighted center-of-mass algorithm^56^. The ratio of specific torque to bead displacements was computed and expressed as the cell stiffness in units of Pascal per nm (Pa/nm).

### Cell proliferation assays

Cellular proliferation was measured by live cell counting using an automated cell counter (Countess Cell Counter, Invitrogen), as well as Cell Counting Kit-8 (CCK-8, Sigma) and the Click-iT EdU Alexa Fluor 647 assay kit (Thermo Fisher). For live cell count and CCK-8 assays, ASM cells were seeded on 6-well plates and maintained in complete DMEM-Ham’s F-12 medium supplemented with 10% fetal bovine serum (FBS). After 24 h, SCFAs were added, and cells were allowed to grow for 6 days in complete media containing 10% FBS. Live cell count was performed on days 1, 4 and 6 following SCFAs treatments.

For EdU cell proliferation assay, cells were first seeded on fibronectin-coated glass coverslips in complete media at 40% confluence and allowed to adhere for 2 h. This was followed by serum starvation for 18 h. Samples were then placed in complete media with the indicated treatments (i.e. untreated, formate, acetate, propionate, and butyrate) in the presence of 5-ethynyl-2′-deoxyuridine (EdU; 10^−5^ mol/L) for 24 h. All experiments were performed in triplicate. After 24 h, samples were rinsed with sterile PBS, fixed and permeabilized. EdU positive nuclei were labeled with Alexa Fluor 647, and all nuclei were labeled with DAPI. Samples were mounted and imaged at x10 magnification (Nikon 80i Epifluorescent microscope equipped with a CoolSnap HQ camera): four non-overlapping fields were imaged per sample. Data analysis was performed using the NIS Elements BR software. The object count function was used to identify Alexa Fluor 647 positive (proliferating cells) and DAPI positive (total cells) nuclei. The ratio of Alexa Fluor 647 positive and DAPI positive nuclei was calculated to obtain the fraction of proliferating cells.

### Detection of ASM ORs

Total RNA was extracted from human ASM cells using the RNeasy Mini Kit according to the manufacturer’s protocols (Qiagen). After DNAse treatment (Qiagen), 1 μg of purified total RNA was used to synthesize cDNA by reverse transcription using iScript cDNA Synthesis Kit (BioRad). As a negative control, mock-reverse transcription samples were prepared by replacing the iScript reverse transcriptase enzyme in the reaction mixture with an equal volume of water. PCR was performed using HotStarTaq Master Mix (Qiagen) following standard thermocycling conditions. To detect ORs in human ASM, we used degenerate human OR primers, PC1 and PC2, as described in ref. 57. These OR were cloned and sequenced. In addition, we performed RT-PCR using gene specific primers for each of the identified ORs. The gene specific OR primers were designed by using the NCBI Primer Blast PCR primer designer tool. The sequences of the gene specific primers are listed in Supplementary Figure 1. Human genomic DNA was used to test the gene specific primers and optimize PCR working conditions. We also ran mock RT-PCR reactions in parallel with all RT reactions, and sequenced all amplified bands of expected size to confirm identity.

### Real time-PCR

For these studies, cDNA was generated using random hexamer primers and SuperScript II Reverse Transcriptase (Applied Biosystems), and real-time PCR was performed using TaqMan Universal PCR Master Mix, fluorogenic probes, and oligonucleotide primers. Unless otherwise stated, we used 2^−∆∆Ct^ method to calculate the relative fold change (RFC) of transcripts normalized to a house-keeping gene (*GAPDH*).

### OR trafficking and surface localization

ORs identified in ASM by degenerate primer PCR were cloned into the PME18S mammalian expression vector containing N terminal Lucy, Flag and Rho tags as previously described^34^. These constructs were then transfected into HEK 293T cells with and without the chaperone proteins RTP1S and Ric8b. The ability of each construct to traffic and incorporate into the plasma membrane was determined by surface immunofluorescence as previously described^34^. This consisted of staining live non-permeabilized cells with rabbit polyclonal anti-Flag antibody (Sigma) at 4 °C. In order to label the internal OR population, cells were then fixed with 4% paraformaldehyde, permeabilized with 0.3% Triton-X 100, and stained with a monoclonal anti-Flag antibody (Sigma). Primary antibodies were detected using Alexa Fluor goat anti-rabbit 555 (for surface ORs) and goat anti-mouse 488 (for internal ORs) secondary antibodies (Invitrogen).

### Luciferase assay

An OR51E2, Olfr78 or pcDNA4 construct was transfected into HEK 293T cells with a CREB-dependent luciferase (*firefly)* and a constitutively active luciferase (*renilla*). OR51E2 and Olfr78 constructs were Lucy-Rho-Flag tagged and co-transfected with RTP1S and Ric8b, as previously described^34^. When stimulated by a ligand, ORs signal though the cAMP second messenger. The rise in cAMP within the cell drives an increase in *firefly* expression, which can be measured using a FLUOstar Omega microplate reader (BMG labtech). *Firefly* luciferase levels are measured relative to the constitutively active *renilla* allowing for normalization of the assay to transfection efficiency and variation in cell number. Here transfected cells were plated on 96-well tissue culture plates for ~20 h prior to the exposure to chemical ligands for 4 h. After 4 h, cells were lysed, and *firefly/renilla* ratios were measured in triplicate, as described previously^26^,^34^. For graphs comparing data from more than one construct (i.e., Olfr78 vs. pcDNA4), each construct/condition is normalized to its’s own control (0 mM).

### CRISPR-Cas9

Two single-guide RNAs (sgRNAs) targeting the human *OR51E2* gene (Supplementary Fig. 6a) and three sgRNAs targeting the murine *Olfr78* gene (Fig. 4a) were designed with the assistance of the CRISPR Design Tool from the Zhang laboratory at the Massachusetts Institute of Technology (http://crispr.mit.edu). The sgRNA oligos were cloned into lentiCRISPRv2 plasmid (Addgene) as previously described^58^. Each plasmid containing inserted sgRNA sequence was verified using Sanger sequencing. To make lentivirus, the constructed lentiCRISPRv2 plasmids were co-transfected into HEK 293T cells with packaging plasmids pMD2.G and psPAX2 (Addgene) using calcium phosphate transfection method in P100 dishes. Packaged lentivirus was then transduced into ASM cells using T25 flask or 6-well plate, followed by puromycin selection for 2–3 days. The surviving cells were maintained for another 3 days before isolation of the genomic DNA or proteins.

### Sanger sequencing

To determine the genotype and the genome editing efficiency of human *OR51E2*, PCR products amplified from cell population by genomic DNA spanning the CRISPR RNA (crRNA) target site were cloned into T vector pMD20 (Takara Bio Inc., Japan). Sanger sequencing was performed on 33 single bacterial colonies (Supplementary Fig. 6b). All showed specific ‘indel’ mutations. Thirty one (94%) were of the deletion type showing mostly frameshift mutations near the gRNA cut site. Two (6%) were of the insertion type with one showing a frameshift mutation. For the genome editing efficiency of mouse *Olfr78*, we ran western blots using a validated antibody for Olfr78^59^.

### Western blotting

ASM cells in culture were first washed with PBS, lysed with SDS Protein-Gel Loading Solution (Quality Biological, Inc.), and applied on SDS-PAGE. The separated proteins were then transferred to nitrocellulose membranes. Membranes were blocked with 5% nonfat dry milk in Tris Buffered Saline with 0.1% Tween, and incubated with the primary antibody followed by HRP-conjugated secondary antibody. HRP-labelled bands were detected using an enhanced chemiluminescence (ECL) kit (Pierce) according to manufacturer’s recommendations. Full-length gels and blots are presented in Supplementary Figure 7.

### Amgen lung cell dataset

Primary human bronchial epithelial cells, bronchial smooth muscle cells, lung fibroblasts and microvascular endothelial cells from normal or diseased patients were obtained from Lonza (Basel, Switzerland). Low passage cells (passage number 4–6) were used for RNA-Seq, and cells were grown to ~80% confluency before RNA isolation. Bronchial epithelial cells from 3 normal, 3 COPD and 3 asthma donors were grown submerged in BEBM Base Media with BEGM Bullet Kit (Lonza). Primary human bronchial smooth muscle cells from 3 normal, 3 COPD and 3 asthma donors were cultured in SmBM Base Media supplemented with SmGM^TM^-2 Bullet Kit (Lonza). Lung fibroblasts from 3 normal, 4 COPD and 4 asthma donors were cultured in FBM Base Media supplemented with FGM-2 Bullet Kit (Lonza). Lung fibroblasts from seven idiopathic lung fibrosis (IPF) patients were obtained from ATCC or Asterand. IPF cells LL29 (AnHa) (ATCC^®^ CCL-134™) and LL97A (AlMy) (ATCC^®^ CCL-191™) were grown in Hanks F12 Media with 15% FBS were and used at passage 5 and 10, respectively, for RNA-Seq.

### RNA-Sequencing

The Amgen cDNA libraries were prepared from total RNA isolated using Mirvana miRNA isolation kits (Ambion, Grand Island, NY) with on-colum DNase treatment as described previously^60^. Total RNA quality and concentration was determined using Bioanalzyer (Agilent, Santa Clara, CA) and NanoDrop (ThermoScientific, Wilmington, DE) instruments. cDNA was prepared using a modified protocol based on the Illumina TruSeq RNA Sample Preparation Kit (Illumina, San Diego, CA) and published methods for strand-specific RNA-Seq^61^,^62^. After size-selection of libraries (Pippen Prep; SAGE Biosciences, Beverly, MA), dUTP-containing cDNA strands were eliminated by digestion with USER enzymes (New England BioLabs, Ipswich, MA) followed by PCR enrichment for introduction of strand specificity. The enriched cDNA libraries were analyzed in Agilent Bioanalzyer and quantified by Quant-iTTM Pico-Green assays (Life Technologies). The samples were sequenced on the Illumina HiSeq platform (Expression Analysis Inc, Morrisville, NC) using 75 bp paired-end sequencing to a depth of 35–55 million reads.

### RNA-Seq data analysis

The gene model used for the study is the Array Studio (OmiSoft, NC) “OmicsoftGene20130723” gene model, which was derived from the UCSC database (downloaded on July 23, 2013; see also http://www.arrayserver.com/wiki/index.php?title=Omicsoft_Gene_Model). OR genes were identified using the Huge Gene Nomenclature Committee (HGNC) Olfactory Receptor gene set, downloaded on September 14^th^, 2015 (pseudogenes were excluded; we also individually examined any gene in the gene model with an “OR” prefix). The gene model contained 375 such ORs. As the primary focus of this investigation was OR expression in smooth muscle, we focused on identifying ORs with clear expression in the cultured smooth muscle cell samples from the Amgen lung dataset (see Supplementary Methods). ORs were ranked in order of descending FPKM, and also considered in the context of cross-sample support, as measured by the number of samples with more than one read aligning to the OR. We would like to emphasize that we consider evaluation of OR expression using these datasets to be exploratory, as OR expression is generally low and sensitive to technical considerations such as library depth and complexity and the noise inherent to quantification of low-abundance transcripts. Alignment coverage profiles of the highest ranking ORs (shown in Supplementary Table 1) were individually scrutinized in the Array Studio genome browser to ensure that no obvious anomalies were observed (e.g. pile-ups of non-unique fragments or coverage that was inconsistent with the gene model). As an example, we provide a zoomed out view of OR51E2 RNA-Seq coverage in Supplemental Figure 3, highlighting the robust coverage and high specificity of expression in the region. Please see Supplementary Methods for details on other RNA-Seq datasets employed (i.e. Genotype-Tissue Expression Project; BLUEPRINT Hematopoietic Epigenome Project) and the detailed analysis workflow.

### Statistical analysis

Unless otherwise stated, data are presented as mean ± standard error of the mean. We used unpaired Student’s *t*-tests, and the analysis of variance (ANOVA) with adjusting for multiple comparisons by applying the Bonferroni method. Where indicated, we also applied Student-Newman-Keuls method. To satisfy the distributional assumptions associated with *t*-tests and the ANOVA, stiffness and MSD data were converted to log scale prior to analyses. All analyses were performed using SigmaPlot (Systat Software) or GraphPad Prism (La Jolla, CA), and 2-sided *P* values less than 0.05 were considered significant.

## Additional Information

**How to cite this article**: Aisenberg, W. H. *et al*. Defining an olfactory receptor function in airway smooth muscle cells. *Sci. Rep.* **6**, 38231; doi: 10.1038/srep38231 (2016).

**Publisher’s note:** Springer Nature remains neutral with regard to jurisdictional claims in published maps and institutional affiliations.

## Supplementary Material

Supplementary Figures

Supplementary Methods

Supplementary Table 1
