# Supplementary material for: Defining an olfactory receptor function in airway smooth muscle cells
Source: Sci Rep. 2016 Dec 1;6:38231. doi: 10.1038/srep38231 (PMC5131280; doi:10.1038/srep38231)
Supplement: Supplementary Figures [file srep38231-s1.doc]

Supplementary Figures

Defining an olfactory receptor function in airway smooth muscle cells

William H. Aisenberg^1^,*, Jessie Huang^2^,*, Wanqu Zhu^2^,*, Premraj Rajkumar^1,*^, Randy Cruz^2^, Lakshmi Santhanam^3^, Niranjana Natarajan^1^, Hwan Mee Yong^2^, Breann De Santiago^2^, Jung Jin Oh^2^, A-Rum Yoon^2^, Reynold A. Panettieri^4^, Oliver Homann^5^, John K. Sullivan^6^, Stephen B. Liggett^7^, Jennifer L. Pluznick^1,†^, and Steven S. An^2,8,9,†^

^1^Department of Physiology, Johns Hopkins School of Medicine, Baltimore, MD 21205, USA; ^2^Department of Environmental Health Sciences, Johns Hopkins Bloomberg School of Public Health, Baltimore, MD 21205, USA; ^3^Department of Anesthesiology and Critical Care Medicine, Johns Hopkins School of Medicine, Baltimore, MD 21205, USA; ^4^Institute for Translational Medicine and Science, Rutgers University, New Brunswick, NJ 08901, USA; ^5^Genome Analysis Unit, Amgen Inc., South San Francisco, CA 94080, USA; ^6^Department of Inflammation, Amgen Inc., Thousand Oaks, CA 91320, USA; ^7^Department of Internal Medicine and Molecular Pharmacology and Physiology, and the Center for Personalized Medicine and Genomics, University of South Florida Morsani College of Medicine, Tampa, FL 33612, USA; ^8^Department of Chemical and Biomolecular Engineering, Johns Hopkins University, Baltimore, MD 21205, USA; ^9^Department of Biomedical Engineering, Ulsan National Institute of Science and Technology, Ulsan 689-798, Republic of Korea.

*These authors contributed equally to this work as co-first authors.

†Corresponding authors. E-mails: san3@jhu.edu (S.S.A.); jpluznick@jhmi.edu (J.L.P.)

**Funding:** This work was supported by US National Heart, Lung, and Blood Institute grants HL107361 (to S.S.A.) and HL114471 (to S.S.A., S.B.L., R.A.P.). S.S.A, L.S., and J.L.P. were also supported by the Johns Hopkins University Discovery Award.

**Competing financial interests:** The authors declare no competing financial interests.

| Human ADCY3 | 360 bp | CGGTGGAGAAGGAGAAGCAGAGTGG |
| --- | --- | --- |
|  |  | CCTCCGTTTCCATCCCTGCCGTTGC​ |
| Human Golf | 445 bp | TACCAGCTGATTGACTGTGC |
|  |  | TTGCATATTCTGGGAAATAG |
| **OR1J1** | 600 bp | ACTCAGCACCTAGCCGTCTT |
|  |  | GTTCAACATGGGAGTGACTGCT |
| **OR1J2** | 299 bp | GGGTGGTGGTCATTACCCTG |
|  |  | CCCAAGGGCCTCTTTCATGT |
| **OR1J2 full length** | 942 bp | CTCCAACAAGACGGGCGTGGTGGAATTCATGAGCCCTGAGAACCAGAGCAGCGT |
|  |  | CGCAGAACTGGTAGGTATGGAAGATCCCTCGAGTCACCAAGAGAAAAATGTTGCTCTACTGAAGAGTTTCCCAAGGG |
| **OR6A2** | 266 bp | TGATGCACATTCCTTCGGCT |
|  |  | AGGATCCTGGTGCTGGTACA |
| **OR6A2 full length** | 1384 bp | CTCCAACAAGACGGGCGTGGTGGAATTCATGGAGTGGCGGAACCATAGTGGGAGAGT |
|  |  | CGCAGAACTGGTAGGTATGGAAGATCCCTCGAGCTATACATTTCTGCTAGCTTTCTTGGGGTCAGGATCCTGG |
| **OR2A1** | 356 bp | ATCCTGGGGCTCATCTCACT |
|  |  | ATGAGAACCACATGGGCCAG |
| **OR2Al full length** | 933 bp | CTTTCTCCAACAAGACGGGCGTGGTGGAATTCATGGGGGAAAATCAGACAATGGTCACAGAGTTCC |
|  |  | CGCAGAACTGGTAGGTATGGAAGATCCCTCGAGTTAGGAATGACTTTCCTTGCCCAGTGCTCTC |
| **OR51E2** | 554 bp | CAGCCATTGACCTGGCCTTA |
|  |  | GAAGGCGAGTACCACACCAA |
| **OR51E2 full length** | 979 bp | TCCAGCTATGAGTTCCTGCAA |
|  |  | TGTTAAGGGTCACTTGCCTCC |

**Supplementary Figure 1: *Nucleotide sequences of the primers used to detect canonical olfactory signaling machinery from the cDNA of human ASM.*** The table provides the nucleotide sequences of both the forward and reverse primers used to detect ORs, G_olf_ and AC3 in the human ASM. The expected size of the bands noticed in the PCR reactions is also listed.

**Supplementary Figure 2: *Expanded visualization of OR RNA-Seq expression****.* Expression is represented as described for Fig. 2 of the primary manuscript, but expanded to include raw RNA-Seq read counts (right columns) and BLUEPRINT immune cell data.

**Supplementary Figure 3: *RNA-Seq coverage of OR51E2 and neighboring OR genes.*** Strand-specific RNA-Seq read coverage of OR51E2, and to a lesser extent OR51E1, does not extend to neighboring OR genes in the OR-dense locus of chromosome 11. All genes shown with the “OR” prefix are olfactory receptor genes. Data are shown for Amgen lung panel cell types (excluding the endothelial cells due to low sample count and low coverage). The visualization is generated using the Array Studio gene browser (Omicsoft, NC) with “intron-trimming” enabled to condense the view by eliminating inter-genic and intra-exonic chromosome regions.

**Supplementary Figure 4:** ***Effects of lactate on luciferase-based reporter assay and single-cell mechanical analyses.* (a)** Dose-response curves for Olfr78 (the murine ortholog of OR51E2) to acetate, propionate and lactate. HEK 293T cells transfected with empty vector (pcDNA4) served as negative controls. Each construct/condition was normalized to it’s own control (0mM). Whereas Olfr78 significantly responded to acetate (at all doses greater 1mM) and propionate (at all doses greater than 0.5mM), Olfr78 significantly responded to lactate at 50mM (ANOVA with Student-Newman-Keuls, *, P < 0.05 vs. 0mM for the same condition). The EC_50_ for acetate was 2.43mM (similar to previously published values of 2.35mM (Change et al., 2015) and 2.01mM (Pluznick et al., 2013). The EC50 for lactate was 21.39mM. (**b**) Although OR51E2 exhibited a clear dose-response to both acetate and propionate (*, P < 0.05 vs. 0mM within a condition by ANOVA with Student-Newman-Keuls), OR51E2 failed to respond to lactate. **(c)** Computed MSD at 300s for human ASM cells treated for 4h with increasing concentrations of lactate. Data are presented as Geometric Mean + 95% CI (n=296-359 individual beads measurements). *P < 0.05 compared with untreated cells using ANOVA analysis. **(d)** Computed MSD at 300s for mouse ASM cells treated with 50mM lactate for 4h and 24h. Data are presented as Geometric Mean + 95% CI (4h, n=343-350; 24h, n=268-320 individual beads measurements). [Unpaired Student’s *t*-test].

**Supplementary Figure 5: *Single-cell analyses on ASM stiffness and cytoskeletal remodeling.* (a)** Human ASM cells were treated for 4h with or without 10mM SCFAs, and dynamic changes in ASM stiffness measured in response to 10μM histamine. For each individual human ASM cell, baseline stiffness was measured for the first 60s, and after drug addition stiffness was continuously measured for the next 240s. For each cell, stiffness was normalized to its baseline stiffness prior to the agonist stimulation. Data are presented as Mean + SE (n=449-923 cells). **(b)** 24h exposures to acetate and propionate decreased baseline cell stiffness (fraction of the untreated cells). Data are presented as Mean + SE (n=265-286 cells). *P < 0.05 compared with untreated cells using ANOVA analysis. **(c-d)** Computed MSD for cells treated for 4h (**c**) or 24h (**d**) with increasing concentrations of acetate (n=266-572) and propionate (n=89-547). Data are presented as Mean + SE.

**Supplementary Figure 6: Genome editing of *OR51E2* in primary human ASM cells. (a)** A schematic diagram of human *OR51E2* locus and its exons; two target sites in exon 3 are shown below, indicating the CRISPR N20NGG target sites. (**b**) To determine the genotype and the genome editing efficiency, we used Sanger sequencing of the PCR products amplified from cell population by genomic DNA spanning the gRNA target site. The PCR products were also cloned, and Sanger sequencing was performed on 33 single bacterial colonies. All showed specific ‘indel’ mutations. (**c**) Computed MSD at 300s for control and OR51E2 KO cells in response to 24h exposures to SCFAs. Data are presented as Mean + SE (Control, n=425-512; OR51E2 KO, n=485-516 individual beads measurements for each treatment). *P < 0.05 compared with untreated cells using ANOVA analysis.


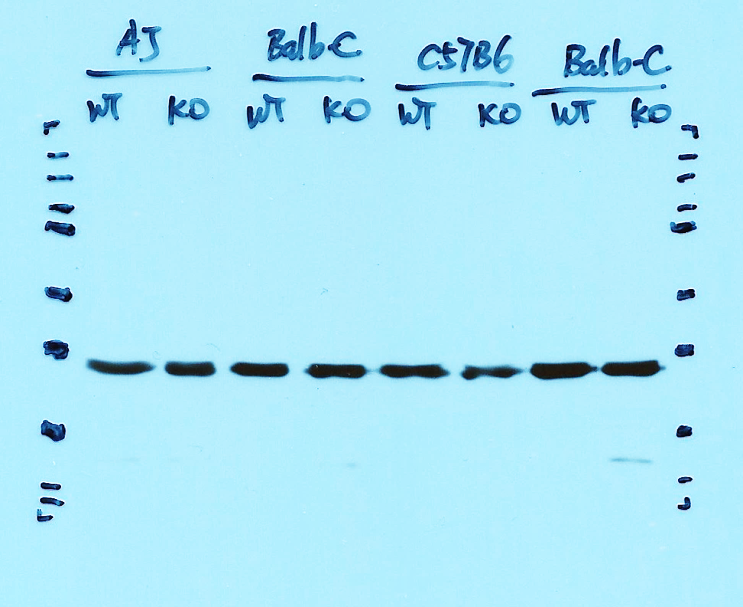


**250 kD**

**150**

**100**

**75**

**50**

**37**

**20**

**15**

**25**

WT

KO

WT

KO

WT

KO

AJ

BALB/c

C57BL/6

IB: GAPDH


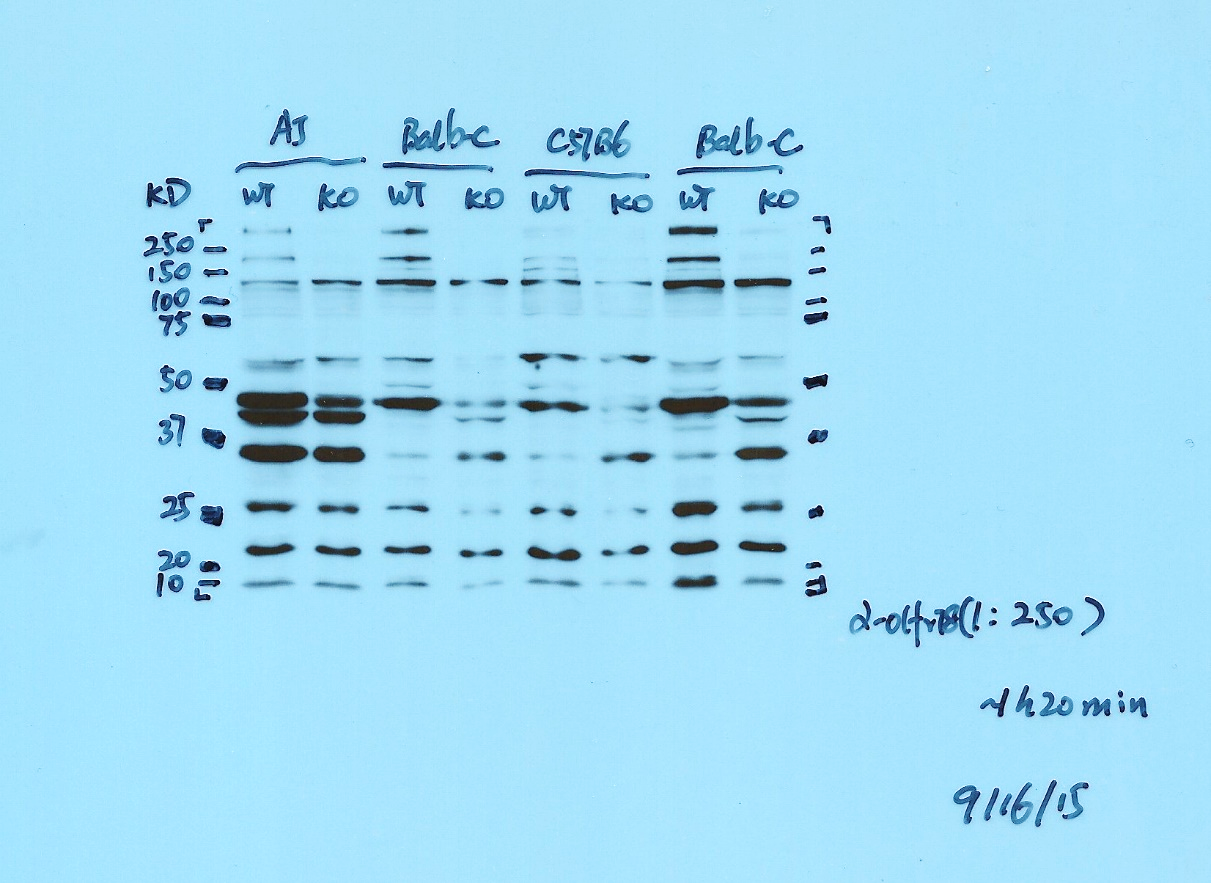


**250 kD**

**150**

**100**

**75**

**50**

**37**

**20**

**15**

**25**

WT

KO

WT

KO

WT

KO

AJ

BALB/c

C57BL/6

IB: Olfr78

**a**


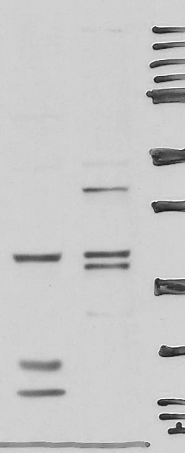


IB: FLAG

Low exposure

**250 kD**

**150**

**100**

**75**

**50**

**37**

**25**

**20**

**15**

**WT**

**Flag-OR51E2**

IB: FLAG

High exposure


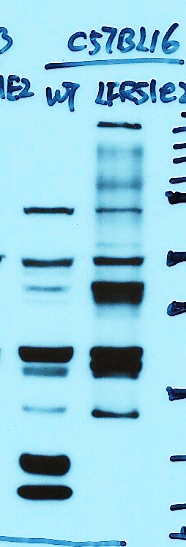


**250 kD**

**150**

**100**

**75**

**50**

**37**

**25**

**20**

**15**

**WT**

**Flag-OR51E2**


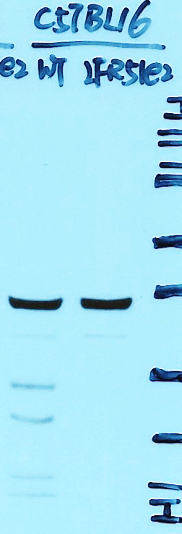


IB: GAPDH

**250 kD**

**150**

**100**

**75**

**50**

**37**

**25**

**20**

**15**

**WT**

**Flag-OR51E2**

**b**

**Supplementary Figure 7:** ***Uncropped images of western blots presented in Figure 4.*** (**a**) Full-length blots of Olfr78 expressed in primary ASM cells derived from inbred mouse strains as shown in **Figure 4c**. (**b**) Full-length blots of ASM cells derived from C57BL/6 transfected with or without FLAG-tagged full-length construct encoding human OR51E2. OR51E2 protein expression was detected by anti-Flag antibody at both low and high exposure time. **Figure 4e** shows the high exposure gel blot. The expected band is ~36 kDa.

**Supplementary Figure 8:** ***Physiologic effects of acetate and propionate are not mediated via GPR41/43.*** (**a**) Human ASM expressed both GPR41 and GPR43 as detected by PCR. (**b-c**) Knockdown of GPR41 and GPR43 with a short hairpin RNA complementary to GPR41 and GPR43 mRNAs. Data are presented as Mean + SE (n=3 independent measurements). (**d-e**) Live cell count after 6 days in culture with acetate and propionate (n=2 independent measurements). Data are presented as Mean + SE. Both acetate and propionate decreased proliferation of GPR41/43-silenced ASM cells. *P < 0.05 compared with respective untreated cells using unpaired Student’s *t*-test.
