# Supplementary material for: Defining an olfactory receptor function in airway smooth muscle cells
Source: Sci Rep. 2016 Dec 1;6:38231. doi: 10.1038/srep38231 (PMC5131280; doi:10.1038/srep38231)
Supplement: Supplementary Methods [file srep38231-s2.doc]

Supplementary Methods

Defining an olfactory receptor function in airway smooth muscle cells

William H. Aisenberg^1^,*, Jessie Huang^2^,*, Wanqu Zhu^2^,*, Premraj Rajkumar^1,*^, Randy Cruz^2^, Lakshmi Santhanam^3^, Niranjana Natarajan^1^, Hwan Mee Yong^2^, Breann De Santiago^2^, Jung Jin Oh^2^, A-Rum Yoon^2^, Reynold A. Panettieri^4^, Oliver Homann^5^, John K. Sullivan^6^, Stephen B. Liggett^7^, Jennifer L. Pluznick^1,†^, and Steven S. An^2,8,9,†^

^1^Department of Physiology, Johns Hopkins School of Medicine, Baltimore, MD 21205, USA; ^2^Department of Environmental Health Sciences, Johns Hopkins Bloomberg School of Public Health, Baltimore, MD 21205, USA; ^3^Department of Anesthesiology and Critical Care Medicine, Johns Hopkins School of Medicine, Baltimore, MD 21205, USA; ^4^Institute for Translational Medicine and Science, Rutgers University, New Brunswick, NJ 08901, USA; ^5^Genome Analysis Unit, Amgen Inc., South San Francisco, CA 94080, USA; ^6^Department of Inflammation, Amgen Inc., Thousand Oaks, CA 91320, USA; ^7^Department of Internal Medicine and Molecular Pharmacology and Physiology, and the Center for Personalized Medicine and Genomics, University of South Florida Morsani College of Medicine, Tampa, FL 33612, USA; ^8^Department of Chemical and Biomolecular Engineering, Johns Hopkins University, Baltimore, MD 21205, USA; ^9^Department of Biomedical Engineering, Ulsan National Institute of Science and Technology, Ulsan 689-798, Republic of Korea.

*These authors contributed equally to this work as co-first authors.

†Corresponding authors. E-mails: san3@jhu.edu (S.S.A.); jpluznick@jhmi.edu (J.L.P.)

**Running Title:** Functional expression of OR51E2 in ASM

**Funding:** This work was supported by US National Heart, Lung, and Blood Institute grants HL107361 (to S.S.A.) and HL114471 (to S.S.A., S.B.L., R.A.P.). S.S.A, L.S., and J.L.P. were also supported by the Johns Hopkins University Discovery Award.

**Competing financial interests:** The authors declare no competing financial interests.

## RNA-Seq datasets. Three RNA-Seq datasets were employed to cross-validate ORs and more broadly survey tissue/cell distribution of OR expression:

## Genotype-Tissue Expression Project (GTEx^1^; [**http://www.gtexportal.org/home/**](http://www.gtexportal.org/home/)): 30 different human tissue/cell types (tissues and sample counts are listed in Figure 2a of the primary manuscript).

## BLUEPRINT Hematopoietic Epigenome project (BLUEPRINT; [**http://www.blueprint-epigenome.eu/**](http://www.blueprint-epigenome.eu/)): 29 human immune cell types, as listed in Supplementary Figure 2. See below for data use statement.

## Amgen Lung Cell Dataset (unpublished and described in the Methods): Cultured endothelial, epithelial, fibroblast, and smooth muscle cells.

The GTEx and BLUEPRINT RNA-Seq data were obtained as FPKM and raw read count values from Omicsoft (Cary, NC) through their Array Studio “Land” platform, and normalized as described below for the Amgen dataset. The GTEx dataset was downloaded on December 3, 2015, and the BLUEPRINT dataset was downloaded on October 26^th^, 2015.

The Amgen dataset was processed by Amgen scientists using OmicSoft’s “Oshell” tool (v8.0.3.99). The full data-processing script is provided in a section below; it uses the same parameters as the Omicsoft-processed GTEx and BLUEPRINT data. The FPKM values in all three datasets were generated using Omicsoft’s implementation of the RSEM^2^ algorithm (with Omicsoft’s “UTR trimming” modification disabled). All FPKM data were normalized using upper-quartile normalization^3^ (i.e. multiplying every FPKM value by a correction value such that the 75^th^ percentile gene value of each sample is 10). Only genes with at least one transcript of length > 500bp were used for calculation of the correction value (to avoid undue influence of small RNAs on the distribution).

## RNA-Seq alignment and quantification Oshell Script. The script below shows the parameters used to process the Amgen lung cell RNA-Seq data using Omicsoft’s Oshell tool (v8.0.3.99). Modules involving extended RNA-Seq analysis (e.g. QC, fusion/junction/mutation-detection) are omitted for brevity.

Begin Macro;

@ProjectFolder@ <OUTPUT_PATH>;

@ThreadNumber@ 2;

@ReferenceLibrary@ Human.B37.3;

@GeneModel@ Human.B37.3_OmicsoftGene20130723;

@ProjectName@ <NAME>;

@PairedEnd@ true;

@CompressBam@ true;

@CompressBamQualityCutoff@ 12;

@GreedyAlignment@ false;

@FastqFilePaths@ <FASTQ_FILE_PATHS>;

@AlignmentName@ alignment;

@OsaVersion@ 4;

@FileSeparator@ "/";

@CountGeneName@ count_gene;

@ExportDirName@ exported_data;

End;

Begin NewProject;

File "@ProjectFolder@@FileSeparator@@ProjectName@.osprj";

Options /Distributed=true;

End;

Begin MapRnaSeqReadsToGenome /Namespace=NgsLib;

Files

"@FastqFilePaths@";

Reference @ReferenceLibrary@;

GeneModel @GeneModel@;

Options /BamSubFolder=@AlignmentName@ /Version=@OsaVersion@ /ParallelJobNumber=1 /ThreadNumber=@ThreadNumber@ /IndexMode=14Mer /FileFormat=FASTQ /QualityEncoding=Automatic /CompressionMethod=Gzip /ReportCutoff=10 /ExcludeNonUniqueMapping=false /ExcludeUnmappedInBam=false /WriteReadsInSeparateFiles=false /GenerateSamFiles=false /CompressBam=@CompressBam@ /CompressBamQualityCutoff=@CompressBamQualityCutoff@ /PairedEnd=@PairedEnd@ /AutoPenalty=true /FixedPenalty=2 /DetectIndels=true /IndelPenalty=2 /MaxMiddleInsertionSize=10 /MaxMiddleDeletionSize=10 /MaxEndInsertionSize=10 /MaxEndDeletionSize=10 /MinDistalEndSize=3 /ExpectedInsertSize=300 /InsertSizeStandardDeviation=40 /Greedy=@GreedyAlignment@ /InsertOnSameStrand=false /InsertOnDifferentStrand=true /ExpressionMeasurement=None /Add1=false /SearchNovelExonJunction=true;

Output @AlignmentName@;

End;

Begin ReportGeneTranscriptCounts /Namespace=NgsLib;

Project @ProjectName@;

Data @ProjectName@\\@AlignmentName@;

Reference @ReferenceLibrary@;

GeneModel @GeneModel@;

Options /OutputFolder="@ProjectFolder@@FileSeparator@@ProjectName@@FileSeparator@@AlignmentName@" /ExpressionMeasurement=RPKM+Count /CountFragments=true /ExcludeMultiReads=false /UseEffectiveTranscriptLength=false /CountStrandedReads=true /CountReverseStrandedReads=true /Add1=false /ThreadNumber=@ThreadNumber@;

Output @CountGeneName@;

End;

Begin SaveProject;

Project @ProjectName@;

File "@ProjectFolder@@FileSeparator@@ProjectName@.osprj";

End;

Begin ExportView;

OutputFolder "@ProjectFolder@@FileSeparator@@ExportDirName@";

Output;

End;

Begin CloseProject;

Project @ProjectName@;

End;

## BLUEPRINT data use statement. This study makes use of data generated by the BLUEPRINT Consortium. A full list of the investigators who contributed to the generation of the data is available from www.blueprint-epigenome.eu. Funding for the project was provided by the European Union's Seventh Framework Programme (FP7/2007-2013) under grant agreement no 282510–BLUEPRINT.

**REFERENCES**

1. Consortium, G.T. The Genotype-Tissue Expression (GTEx) project. *Nat Genet* **45**, 580-585 (2013).

2. Li, B. & Dewey, C.N. RSEM: accurate transcript quantification from RNA-Seq data with or without a reference genome. *BMC bioinformatics* **12**, 323 (2011).

3. Robinson, M.D. & Oshlack, A. A scaling normalization method for differential expression analysis of RNA-seq data. *Genome biology* **11**, R25 (2010).
